# Store-operated Ca^2+^ entry is activated by every action potential in skeletal muscle

**DOI:** 10.1038/s42003-018-0033-7

**Published:** 2018-04-19

**Authors:** Xaver Koenig, Rocky H Choi, Bradley S Launikonis

**Affiliations:** 1 School of Biomedical Sciences, The University of Queensland, Brisbane, QLD 4072 Australia; 2 Center for Physiology and Pharmacology, Medical University of Vienna, Schwarzspanierstrasse 17, 1090 Wien, Austria

## Abstract

Store-operated calcium (Ca^2+^) entry (SOCE) in skeletal muscle is rapidly activated across the tubular system during direct activation of Ca^2+^ release. The tubular system is the invagination of the plasma membrane that forms junctions with the sarcoplasmic reticulum (SR) where STIM1, Orai1 and ryanodine receptors are found. The physiological activation of SOCE in muscle is not defined, thus clouding its physiological role. Here we show that the magnitude of a phasic tubular system Ca^2+^ influx is dependent on SR Ca^2+^ depletion magnitude, and define this as SOCE. Consistent with SOCE, the influx was resistant to nifedipine and BayK8644, and silenced by inhibition of SR Ca^2+^ release during excitation. The SOCE transient was shaped by action potential frequency and SR Ca^2+^ pump activity. Our results show that SOCE in skeletal muscle acts as an immediate counter-flux to Ca^2+^ loss across the tubular system during excitation-contraction coupling.

## Abstract (web summary)

Xaver Koenig et al. demonstrate the physiological activation of store-operated calcium entry in muscle, pointing to a role for this mechanism. The resulting influx of calcium into the cytoplasm is shaped by action potential frequency and calcium pump activity.

## Introduction

Store-operated Ca^2+^ entry (SOCE) is a mechanism that is slowly activating in most cells, where stromal interacting molecule 1 (STIM1) molecules sense Ca^2+^ depletion in the internal Ca^2+^ store and migrate to plasma membrane contact sites to trigger Ca^2+^ influx through Orai1 channels^1^. In contrast, SOCE has evolved to become a fast mechanism in skeletal muscle, probably in parallel with the evolution of the highly organised membrane structures that support the rapid transients of Ca^2+^ in the fibre evoked by action potentials^2^. In muscle, the surface plasma membrane of the fibre has regular invaginations, to form the tubular (t-) system. The dominant element of the t-system, the transverse tubules reach each sarcomere to form junctions with the sarcoplasmic reticulum (SR) terminal cisternae^3^ where the transverse tubular dihydropyridine receptor (DHPR)/L-type Ca^2+^ channel directly activates the SR ryanodine receptor (RyR) to rapidly activate Ca^2+^ release^4^. This process is known as excitation-contraction coupling (EC coupling) and provides the basis for muscle contraction.

While the presence of a robust and fast SOCE in muscle is clear, the physiological activation and primary roles of SOCE in this tissue remain elusive^2,5^. The biophysical properties of SOCE in muscle^2^ suggest its physiological role can be an immediate one, related to compensatory Ca^2+^ movements during action potential-evoked Ca^2+^ release. Knowledge of the physiological patterns of store-dependent influx and the conditions associated with its activation are critical in defining physiological role, which will help in our understanding of SOCE-related myopathies, developmental defects and fibre-type shifts in *STIM1* and *ORAI1* knockout mice^6–10^.

Ca^2+^ within the SR ([Ca^2+^]_SR_) is strongly buffered by calsequestrin and the high-capacity SR Ca^2+^ pumps^11^. This prevents steady state or tonic activation of SOCE in resting fibres^5,12^. Furthermore, the SR strongly buffers Ca^2+^ during EC coupling, preventing deep depletion of the [Ca^2+^]_SR_^13–15^, making it difficult to understand how SOCE is activated physiologically in skeletal muscle, if we continue to assume that a bulk depletion of [Ca^2+^]_SR_ is required for SOCE activation^5^.

We hypothesized that SOCE could be activated during action potential-evoked Ca^2+^ release by local depletion of Ca^2+^ immediately behind the RyRs at the SR terminal cisternae. Local depletions of Ca^2+^ inside the SR would briefly and uniformly dissociate Ca^2+^ from the local population of STIM1^7,16^ to rapidly active SOCE. The rapid transduction of such a signal requires the static junction between the transverse tubules and the SR terminal cisternae membranes, allowing RyR activity to intimately link EC coupling and SOCE^2^. Consistent with this, it has been demonstrated that only the transverse tubules support SOCE; whereas, the longitudinal elements of the t-system that lack proximity to RyRs have become dynamic Ca^2+^-buffers, devoid of SOCE^17^. Here we use a microscopy-based technique with the necessary sensitivity and temporal resolution to measure the occurring Ca^2+^-fluxes across the t-system membrane^18,19^ under physiological stimulation patterns. This approach allowed us to identify that the physiological activation of SOCE is by every action potential-induced Ca^2+^ release, presenting as a phasic Ca^2+^ influx. This finding allows the elucidation of the physiological roles of SOCE in muscle and broadens our understanding of the properties of STIM1 and SOCE more generally.

## Results

### t-system membrane Ca^2+^ fluxes following every action potential

**Fig. 1:**
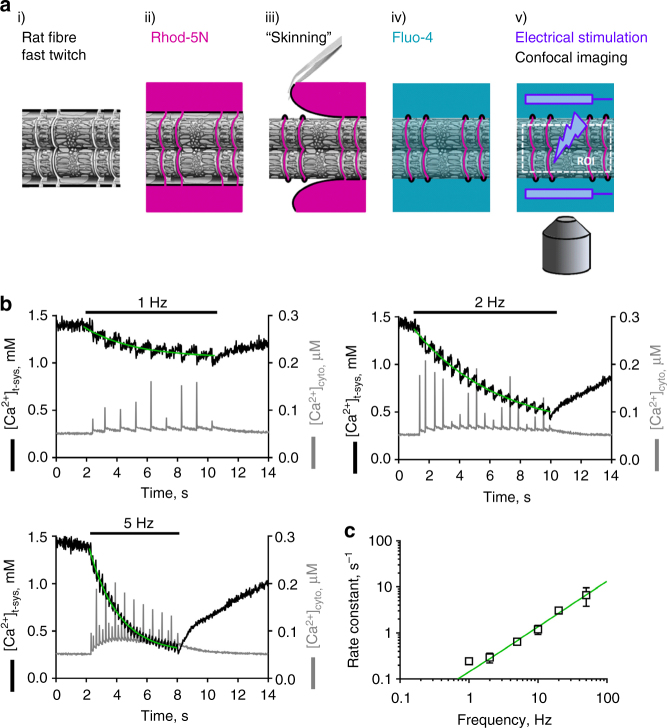
Identifying extracellular Ca^2+^ influx across the t‐system membrane activated by single-action potentials. **a** Schematic representation of the individual protocol steps of the technique. (i) isolation of a single fibre of rat EDL muscle, (ii) incubation of the fibre with the Ca^2+^‐sensitive dye Rhod‐5N, (iii) Mechanical removal of the sarcolemma (skinning) and resulting trapping of the dye within the sealed t‐system, (iv) incubation of the fibre in a physiological salt solution containing the Ca^2+^‐sensitive dye Fluo‐4, (v) electrical field stimulation via platinum electrodes in parallel to the fibre’s long axis, and simultaneous confocal imaging of both dye fluorescence within the region of interest (ROI). **b** Typical recordings of [Ca^2+^]_t‐sys_ (left axes) and [Ca^2+^]_cyto_ (right axes) over time as derived from skinned rat EDL fibres during electrical stimulation. xyt image series of the fluorescent signals of rhod‐5N and fluo‐4 trapped in the t‐system and loaded into the cytosol, respectively, were spatially averaged and calibrated (Methods section). Electrical stimulation at 1, 2 and 5 Hz as indicated was started shortly after beginning of the recording and was continued until a new steady state in the t‐system was reached, unambiguously identifiable by the termination of cytosolic transients. Note that the employed sampling rate of 55 frames s^−1^ is below the Nyquist criterion, which results in an apparent modulation of cytosolic Ca^2+^ transient amplitudes. Green lines represent mono‐exponential fits to the decaying phase of [Ca^2+^]_t‐sys_ upon stimulation. **c** A summary of the respective rate constants as derived from the exponential fits for all tested frequencies. Data are derived from 10 fibres and given as mean ± SEM. EDL, extensor digitorum longus

To resolve Ca^2+^ fluxes across the t-system membrane during action potential-evoked Ca^2+^ release required imaging Ca^2+^ in the cytoplasm and t-system with high sensitivity and millisecond temporal resolution. To do this we used mechanically skinned fibres (Fig. 1a, Methods section), in which we trapped the Ca^2+^-sensitive fluorescent dye rhod-5N in the t-system of skinned fast twitch extensor digitorum longus (EDL) fibres from the rat, as previously described^19^. This preparation preserves intact EC coupling^13,20^ and respective Ca^2+^ release and force responses are indistinguishable from those in intact fibres^19,21–23^. The removal of the sarcolemma during the skinning process grants access to the cytosol and allows for the measurement of the intracellular Ca^2+^ levels with the use of a spectrally separate Ca^2+^-sensitive dye from rhod-5N, fluo-4.

Figure 1b is an example of spatially averaged profiles of cytoplasmic fluo-4 and t-system-trapped rhod-5N fluorescence during electrical field stimulation of a skinned fibre at 1, 2 and 5 Hz using the approach described in Fig. 1a. In each of these cases, field stimulation induced a transient rise in cytosolic Ca^2+^ (Fig. 1b, grey trace). The simultaneous recording of rhod-5N fluorescence showed a decline, indicating a concurrent influx of Ca^2+^ from the t-system to the cytoplasm with each action potential (Fig. 1b, black trace). Both signals returned to their respective baseline levels following the cessation of stimulation. Note that the cytoplasmic solution contained 10 mM EGTA with free [Ca^2+^]_cyto_ buffered to 50 nM. The introduction of EGTA as the dominant cytoplasmic Ca^2+^-buffer allows the calculation of the amount of total Ca^2+^ released from the SR (Methods).

Close inspection of the [Ca^2+^]_t-sys_ signals shows a fast decline in t-system Ca^2+^ associated with each action potential-evoked Ca^2+^ release (Fig. 1b). Termination of Ca^2+^ efflux from the t-system allows the signal to recover toward baseline in between individual stimulations pulses, generating a saw-tooth like pattern. As the time for recovery of [Ca^2+^]_t-sys_ became too short at increasing stimulation frequencies, there was a progressive summation of individual [Ca^2+^]_t-sys_ depletions to reduce [Ca^2+^]_t-sys_ in a frequency-dependent manner (Fig. 1b). The overall decline in the [Ca^2+^]_t-sys_ signal during repetitive electrical stimulation could be fitted by an exponential function. A summary of the rate constants at the stimulation frequencies applied is displayed in Fig. 1c. A positive relationship between stimulation frequency and the rate constant of [Ca^2+^]_t-sys_ depletion was observed. At stimulation frequencies of 20 and 50 Hz, the temporal resolution of the recordings (18 ms, Methods section) omitted to resolve individual [Ca^2+^]_t-sys_ depletion events. Still the depletion of [Ca^2+^]_t-sys_ could be fitted by exponential functions (Supplementary Figure 1). The coherence to the observed relationship is consistent with a successful electrical stimulation at these high frequencies and indicates a coupled Ca^2+^ influx from the t-system to the cytoplasm with each action potential under these conditions.

When we evaluated the [Ca^2+^]_t-sys_ depletion, as well as the recovery of the individual steps for the tested frequencies we noticed that individual depletion steps were getting smaller with each applied pulse (Supplementary Figure 2a). We reasoned that this would be due to the decreasing levels of [Ca^2+^]_t-sys_, the driving force for Ca^2+^ entry^24^. When we quantified the change in [Ca^2+^]_t-sys_ (Δ[Ca^2+^]_t-sys_) with respect to [Ca^2+^]_t-sys_ we obtained a strong linear relationship (Supplementary Figure 2b). Thus, the depletion steps and with it the underlying calcium flux were directly proportional to the Ca^2+^ gradient across the t-system (neglecting the much smaller cytosolic Ca^2+^ concentration) suggesting that the observed Ca^2+^ flux was carried by a Ca^2+^-selective ion channel^24^.

In order to improve the temporal resolution of our signal, we performed linescans, accepting a dramatic loss in signal to noise ratio due to the loss of the spatial averaging. To partly restore for the deteriorated signal we averaged at least 10 consecutive linescans aligned by the peak of the action potential-induced Ca^2+^ release. This provided an improvement in temporal resolution to 80 µs. With this approach, we determined the activation of the Ca^2+^ influx during a single-action potential occurred with a time constant of 10 ms (Supplementary Figure 2c and 2d).

### Every action potential activates SOCE

**Fig. 2:**
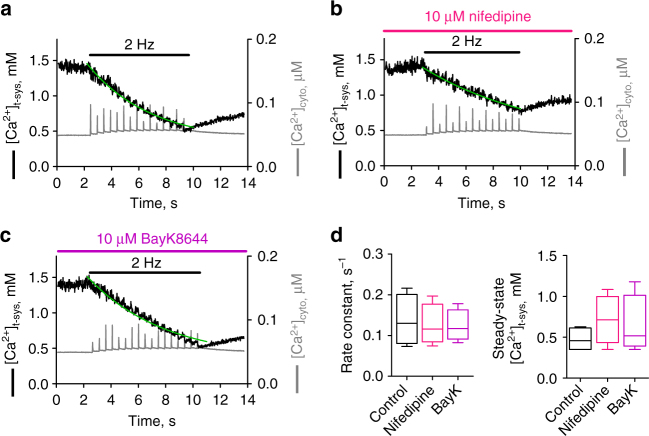
The phasic Ca^2+^ influx is resistant to the DHPR antagonist and agonist, nifedipine and BayK8644, respectively. Depletion of [Ca^2+^]_t-sys_ during 2 Hz stimulation of a skinned fibre under control conditions (**a**), and in the presence of either 10 µM nifedipine (**b**) or 10 µM BayK8644 (**c**). An exponential curve was fitted to the declining phase of the [Ca^2+^]_t-sys_ in each case. The mean rate constant and [Ca^2+^]_t-sys_ steady-state lower plateau levels fitted from four fibres as presented as box and whisker plots in **d**. One-way ANOVA revealed no statistical significance for comparing the rate constants (*p* = 0.8128) and the steady-state levels (*p* = 0.4412), respectively

The known t-system Ca^2+^ fluxes that could be carrying the observed phasic influx during EC coupling are L-type Ca^2+^ channel or SOCE. To determine whether the phasic Ca^2+^ flux was carried by the L-type Ca^2+^ channel or not we applied the known antagonist nifedipine and the agonist BayK8644 and assessed [Ca^2+^]_t-sys_ depletion during trains of action potentials under these conditions. Neither nifedipine, at a close to saturating concentration (10 µM)^25,26^, nor BayK8644 had any effect on the observed phasic [Ca^2+^]_t-sys_ depletion nor its rate of depletion (Fig. 2). These results exclude the possibility that the L-type Ca^2+^ channel is responsible for carrying the observed phasic t-system Ca^2+^ flux.

**Fig. 3:**
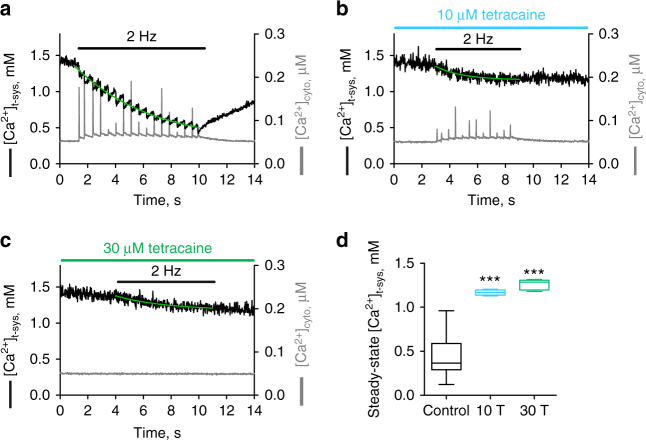
Action potential-activated extracellular Ca^2+^ influx is store‐operated. Representative traces of [Ca^2+^]_cyto_ and [Ca^2+^]_t‐sys_ under control conditions (**a**) or in the presence of either 10 µM tetracaine (**b**) or 30 µM tetracaine (**c**). Green lines represent mono‐exponential fits to the decay in the [Ca^2+^]_t‐sys_ signal during 2 Hz stimulation. **d** The mean [Ca^2+^]_t-sys_ steady-state lower plateaus at the end of the 2 Hz stimulation are presented as box and whisker plots. Data under control conditions (*n* = 7), and in the presence of 10 µM (*n* = 4) and 30 µM tetracaine (*n* = 5) were analysed with One-way ANOVA and Tukey’s post hoc test. ***statistical significance with *p* < 0.0001. Abbreviations on figure: 10 µM tetracaine, 10 T; 30 µM tetracaine, 30 T

Next, to test whether the phasic influx was store-dependent, we examined whether interfering with store-depletion during excitation of the fibre affected the phasic influx. To do this we blocked the RyRs directly, to prevent Ca^2+^ release during field stimulation, using three independent RyR antagonists: tetracaine, ryanodine, and raised free [Mg^2+^]_cyto_. This approach was expected to separate t-system Ca^2+^ fluxes dependent directly on excitability or Ca^2+^ release. First, we tried tetracaine. As expected, 10 µM of the RyR inhibitor tetracaine inhibited the electrically evoked cytosolic Ca^2+^ transients. This concurrently reduced the amount of Ca^2+^ depleted from the t-system (Fig. 3). Raising the tetracaine concentration to 30 µM abolished the cytosolic Ca^2+^ transients and the phasic depletions of [Ca^2+^]_t-sys_ (Fig. 3). Interestingly, although individual depletion steps could not be resolved anymore under these conditions, it was evident that [Ca^2+^]_t-sys_ steady-state values were different before and after electrical stimulation. Thus, electrical stimulation was able to deplete some [Ca^2+^]_t-sys_ even in the absence of any detectable SR Ca^2+^ release. This result is consistent with maintained excitability of the t-system following action potentials in the presence of µM tetracaine and the activation of an apparently tonic, low-amplitude Ca^2+^ current activated by the train of action potentials.

In the next experiments, raised cytosolic [Mg^2+^]_cyto_ and ryanodine were used to inhibit the RyR, both of which are known not to affect t-system excitability^27^. A concentration of 10 µM ryanodine completely abolished SR Ca^2+^ release and the observed phasic depletions of [Ca^2+^]_t-sys_. Consistent with 30 µM tetracaine, a small reduction of the [Ca^2+^]_t-sys_ steady state with stimulation was observed (Supplementary Figure 3). 3 mM free [Mg^2+^]_cyto_ caused a reduction in SR Ca^2+^ release and a reduction in the depletion of [Ca^2+^]_t-sys_ during excitation (Supplementary Figure 4).

We are aware that it is possible for ryanodine and tetracaine to affect the DHPR and voltage-gated Na^+^ channel, respectively. However, the concentrations of ryanodine we have used is well-below the Ki for binding to the DHPR (45 µM)^28^. The presence of 10 µM tetracaine allowed action potential-induced Ca^2+^ release to remain active, though reduced. Furthermore, the use of raised Mg^2+^ as a RyR antagonist is known not to affect t-system excitability^29,30^. Regardless, the presence of the antagonist did not affect the maintenance of excitability as we observed the tonic Ca^2+^ influx in each case, which likely reflects L-type Ca^2+^ channel influx^31^.

Additionally, we examined the possibility that the Ca^2+^ released from the SR into the junctional space per se rather than SR Ca^2+^ depletion caused activation of phasic t-system Ca^2+^ flux. To this end we manipulated the [Ca^2+^] in the junctional space during SR Ca^2+^ release by bathing the fibre in a solution containing 10 mM BAPTA instead of EGTA. BAPTA provides a much faster rate of Ca^2+^ chelation. Under these conditions we observed a suppression of the cytosolic Ca^2+^ transients during EC coupling, as expected, but also observed the persistence of the phasic Ca^2+^ influx from the t-system (Supplementary Figure 5). The rate constant from exponential curves fitted to the decline in [Ca^2+^]_t-sys_ during 2 Hz stimulation and the plateau of [Ca^2+^]_t-sys_ depletion were not significantly different from that in EGTA (two-tailed *t*-tests, *p* = 0.4728 and 0.5833, respectively). This is consistent with depletion of Ca^2+^ from within the SR as the trigger for the phasic t-system Ca^2+^ flux and strongly argues against a mechanism of Ca^2+^-induced Ca^2+^ influx, that could have potentially been carried by members of the TRP family of ion channels

Together, these experiments (Figs. 2, 3 and Supplementary Figures 3-5) clearly demonstrate that the observed rapid [Ca^2+^]_t-sys_ depletion is dependent on the Ca^2+^ depleted from within the SR.

**Fig. 4:**
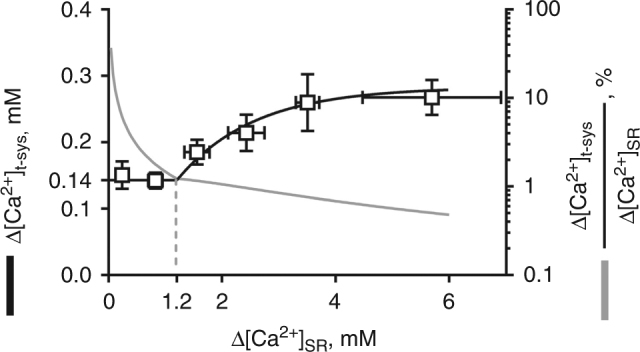
Store dependence of Ca^2+^ influx during electrical stimulation. A summary of the amount of Ca^2+^ entering the cytoplasm from the t‐system in response to the total Ca^2+^ released from the SR following a single-action potential (black line). The ratio of Ca^2+^ entering the cytoplasm from the t‐system and the SR is shown as grey line. Mean ± SEM from six fibres

To be consistent with the phasic flux being SOCE, the degree of SR depletion should be represented in the magnitude of the t-system Ca^2+^ flux^2^. To determine the relationship between the amount of Ca^2+^ released from the SR and the amount of Ca^2+^ depleted from the t-system, we determined the total Ca^2+^ released from the SR under control conditions and under conditions where Ca^2+^ release was either suppressed or facilitated (for example, when Ca^2+^ release was suppressed in tetracaine, Fig. 3, see Methods section for calculations). A summary of this analysis is plotted in Fig. 4. A two component model fitted the data. For very small and non-detectable Ca^2+^ release from the SR (left branch of the graph) there was a constant amount of about 0.14 mM Ca^2+^ depleted from the t-system (with respect to t-system volume). When SR Ca^2+^ release surpassed a threshold of 1.2 mM Ca^2+^ (with respect to SR volume) depletion of the t-system Ca^2+^ increased with increasing Ca^2+^ released from the SR. A maximum plateau was reached at a value of about 0.3 mM (right branch of the graph). The amount of extracellular Ca^2+^ entering the cytoplasm from the t-system was only a small fraction relative to the contribution of the SR during EC coupling. Ca^2+^ released from the SR contributed about 99% to the Ca^2+^ entering the cytoplasm (blue trace, right axis, Fig. 4). Taken together, this data show the presence of a store-independent mechanism (the apparently tonic flux) that can be observed in isolation when no or very little Ca^2+^ was released from the SR and the emergence of a second, store-dependent mechanism (the phasic flux) when a certain threshold of SR Ca^2+^ release was exceeded.

### The SR Ca^2+^ pump restricts SOCE

**Fig. 5:**
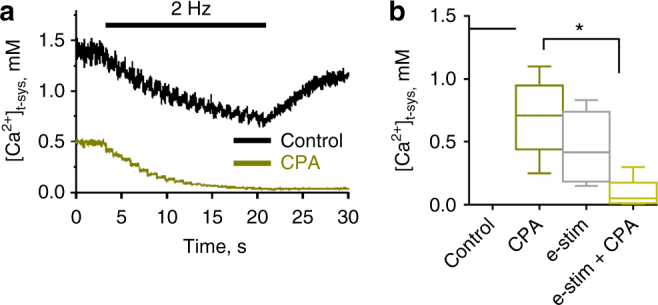
Tonic and phasic SOCE are observed under SR Ca^2+^ pump inhibition. Representative traces of [Ca^2+^]_cyto_ and [Ca^2+^]_t‐sys_ at rest and during 2 Hz field stimulation in the absence (a, top trace) and presence (a, lower trace) of 100 µM CPA. Note that the addition of CPA caused the [Ca^2+^]_t‐sys_ to decline to a new steady‐state level. This new, lower level did not affect the phasic activation of SOCE upon electrical stimulation in the presence of CPA. **b** Summary of [Ca^2+^]_t‐sys_ steady‐state plateau levels before and after 2 Hz electrical stimulation in the absence and presence of CPA. Result from 6 fibres is displayed as a box and whisker plot. One-way ANOVA and Tukey’s post hoc test. *indicates statistical significance with *p* < 0.001

We have proposed that the SR Ca^2+^ pump strongly restricts the activation of SOCE by re-sequestering virtually all of the released Ca^2+^, eliminating the possibility that SOCE refills the SR following EC coupling^5^. To test this we directly assessed SOCE during EC coupling in the presence and absence of a functional SR Ca^2+^ pump. The SR Ca^2+^ pump was poisoned with cyclopiazonic acid (CPA) added to the cytoplasmic solution to inhibit the uptake of Ca^2+^. Figure 5 shows that the addition of 100 µM CPA caused [Ca^2+^]_t-sys_ to be lowered to a new steady level prior to electrical stimulation. This change in [Ca^2+^]_t-sys_ occurred in the absence of a change in the t-system electrochemical gradient for Ca^2+^, indicating that the lowering of [Ca^2+^]_SR_ to a new steady level upon blocking of the SR Ca^2+^ pump caused tonic activation of a subpopulation of SOCE channels. Importantly, the commencement of phasic [Ca^2+^]_t-sys_ depletions with individual electrical stimuli, indicated the additional, rapid recruitment of SOCE channels during the rapid release of Ca^2+^ from SR. This also indicates that [Ca^2+^]_SR_ remained high enough in the presence of CPA prior to electrical stimulation to keep a population of SOCE channels inactive. With cessation of electrical stimulation, the lowered [Ca^2+^]_t-sys_ did not recover in the continued presence of CPA, in contrast to the control (Fig. 5). This indicates that SOCE channels opened phasically during stimulation in the presence of CPA probably remained tonically active as Ca^2+^ re-uptake into the SR was inhibited. The immediate recovery of [Ca^2+^]_t-sys_ following cessation of electrical stimulation in the absence of CPA shows that SOCE channels close or become inactive immediately following the termination of action potential-induced opening of the RyRs.

### SOCE is a counter-flux

**Fig. 6:**
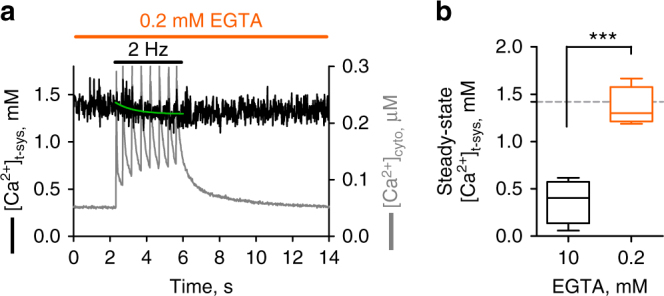
SOCE is an immediate counter‐flux isolated by cytosolic Ca^2+^ buffering. **a** [Ca^2+^]_t‐sys_ and cytoplasmic Ca^2+^ transients in a skinned fibre in the presence of 0.2 mM EGTA during field stimulation of 2 Hz. Note the absence of the phasic [Ca^2+^]_t‐sys_ depletion observed under these conditions, in contrast to cytosolic Ca^2+^‐buffering with 10 mM EGTA (Fig. 1). **b** Summary of steady‐state [Ca^2+^]_t‐sys_ in skinned fibres following 2 Hz field stimulation in the presence of 0.2 and 10 mM EGTA. Result from 4 fibres is presented as a box and whisker plot. Paired two-tailed Student’s *t*-test, ***indicating statistical significance with *p* = 0.0005.

While the presence of 10 mM EGTA in the cytoplasmic solution allowed the calculation of the total Ca^2+^ released from the SR, this level of Ca^2+^-buffering far exceeds endogenous levels. Thus we performed experiments under levels more equivalent to physiological buffering, with [EGTA] lowered from 10 to 0.2 mM^22^. Under field stimulation, with 0.2 mM EGTA in the cytoplasmic solution, there was a marked increase and broadening of the cytosolic Ca^2+^ transients. Importantly, the phasic depletions of [Ca^2+^]_t-sys_ observed in 10 mM EGTA (Fig. 1) could no longer be resolved (Fig. 6). In the same fibre, the re-introduction of 10 mM EGTA allowed these fast [Ca^2+^]_t-sys_ depletions to be observed again (Supplementary Figure 6). Under 2 Hz field stimulation, the steady-state depletion of [Ca^2+^]_t-sys_ was only significant in high [EGTA] (Fig. 6b) (two-tailed *t*-test, *p* = 0.0005). This suggests that high-EGTA suppresses the rise of cytoplasmic [Ca^2+^]_cyto_ to critically reduce the amount of Ca^2+^ able to bind to t-system Ca^2+^-pump binding sites during Ca^2+^ release to reduce Ca^2+^ extrusion. In contrast, under weakly buffered conditions, the higher [Ca^2+^]_cyto_ allows the t-system Ca^2+^ fluxes to be bidirectional and effectively nullify each other. Taken together, the presence of high EGTA provides Ca^2+^-buffering in the junctional space to reduce the ability of the t-system to extrude Ca^2+^, thereby isolating SOCE and the tonic influx of Ca^2+^ during a train of action potentials that results in the observed [Ca^2+^]_t-sys_ depletion (Figs. 1–6)^19^.

## Discussion

The physiological activation mechanism and a primary physiological role of SOCE in skeletal muscle have not been defined, even though the molecular identities and biophysical properties of SOCE have been described in some detail^2,7–9^. The main obstacle in determining the physiological activation and role of SOCE in muscle has been the lack of direct measurements of SOCE during EC coupling under physiological conditions, which we have addressed (Fig. 1). Using this approach we were able to observe a phasic influx of Ca^2+^ from the t-system lumen with every action potential at all stimulation frequencies tested here. The pathway responsible for the Ca^2+^ influx was established as predominantly store-dependent channels by showing (i) a dependence on the amount of Ca^2+^ depleted from the SR (Fig. 4), (ii) resistance to the L-type Ca^2+^ channel antagonist and agonist, nifedipine and BayK8644 (Fig. 2), (iii) an elimination by the blockade of SR Ca^2+^ depletion with three independent RyR antagonists during excitation (Fig. 3 and Supplementary Figures 3, 4), (iv) fast kinetics consistent with a high off-rate of Ca^2+^ for STIM1 (and inconsistent with the slow activation of the L-type Ca^2+^ channel) (Fig. 1), (v) a linear dependence on the t-system Ca^2+^ gradient (Supplementary Figure 2b), and (vi) persistence in the presence of either cytoplasmic EGTA or BAPTA (Supplementary Figure 5). In the absence of Ca^2+^ release, repetitive field stimulation did allow the isolation of an additional mode of slowly activating, apparently tonic Ca^2+^ influx during repetitive action potentials consistent with L-type Ca^2+^ channel activation^31^ (Fig. 3). Changing the Ca^2+^-buffering capacity of the cytoplasm also allowed the manipulation of the ability of the t-system to extrude Ca^2+^ during SR Ca^2+^ release^19,32^. This exposed a role of rapidly activated SOCE during EC coupling was to act as an immediate counter-flux to t-system Ca^2+^ extrusion to prevent the loss of fibre Ca^2+^ during muscle stimulation.

It has been implied from indirect evidence that SOCE is active during the declining phase of the Ca^2+^ transient of a tetanus^9,15^. However, by directly measuring SOCE during EC coupling we show that this is not the case (Figs. 1–3). The physiological waveform of SOCE influx is phasic, following each action potential. The heavily Ca^2+^-buffered SR rapidly inactivated SOCE (Figs. 1–3). We could only observe a tonic activation of SOCE under non-physiological conditions of a poisoned SR Ca^2+^ pump and hence depleted SR Ca^2+^ stores (Fig. 5). During periods of fibre quiescence the electrochemical gradient for Ca^2+^ across the t-system is sufficient for the passive leak of extracellular Ca^2+^ into the fibre to set the required level of Ca^2+^ inside the SR^5,33^. This is consistent with *STIM1* or *ORAI1* null muscle responding normally to field stimulation at 50 Hz, which implies a normal load of SR Ca^2+^^5,7,9,34^. Furthermore, during prolonged periods of muscle stimulation where the muscle experiences metabolic fatigue, the total amount of Ca^2+^ inside the SR is still significant and is not the limiting factor in Ca^2+^ release^35–37^.

The rapid activation of SOCE in conjunction with action potential-induced Ca^2+^ release means only a relatively small quantum of the total Ca^2+^ held within the SR gets released as the trigger SR Ca^2+^ depletion for Ca^2+^ dissociation from STIM1. The steady level of bulk [Ca^2+^]_SR_ does not drop significantly^13–15^ and cannot be critical to SOCE activation. It must be the Ca^2+^ dropping during release in a locally restricted domain behind the RyR channel pore that is critical for SOCE activation. Thus, we suggest that SOCE is rapidly activated by local depletions of Ca^2+^ at the near membrane inside the SR terminal cisternae following the action potential-coordinated Ca^2+^ release. This is, of course, consistent with a k_off_ Ca^2+^ from STIM1 of probably >2000 s^−1^^38^. Near membrane Ca^2+^ depletion will uniformly occur at all the terminal cisternae of the fibre, where STIM1 and RyRs are likely in close quarters^2,16,24^. Under this condition the bulk [Ca^2+^]_SR_ during twitches and tetani can remain high^13–15^ while SOCE becomes briefly active (Figs. 1–4). Our findings provide further support to the proposal that Orai1 and STIM1 are in prearranged complexes that can be rapidly activated upon Ca^2+^ release^2,16,24,32^. Others have challenged this idea based on the increase of a low-biomolecular fluorescence complementation (BiFC) signal in a resting fibre upon poisoning of the SR Ca^2+^ pump and thorough depletion of SR Ca^2+^^9^. The conclusion was based on the assumption that only the stronger BiFC signal represents prearranged complexes. It is, however, possible that both the full and depleted SR have prearranged Orai1-STIM1 complexes, differing only in absolute number, and therefore BiFC signal.

Our line of argument of pre-coupled Orai1-STIM1 complexes is further supported by the observation that SOCE activated with a time constant of only 10 ms (Supplementary Figure 2c, d). In fact this makes it hard to envision STIM1 and Orai1 physically separated at the junctional membranes, given that respective activation kinetics of uncoupled Orai1-STIM1, as has been demonstrated in multiple non-muscle tissues, lie in the seconds range^1^.

The simultaneous imaging of Ca^2+^ in the cytoplasm and SR lumen has shown that the nadir of Ca^2+^ depletion inside the SR lags behind the closure of RyRs during sparks and action potential-induced Ca^2+^ release by 50 and 30 ms, respectively^13^. To explain the anomaly of SR Ca^2+^ depletion continuing after the cessation of Ca^2+^ release, the source of the Ca^2+^ during these events was proposed to originate from a locally restricted domain near the membrane behind the RyR channel pore whose diffusional properties differ from the bulk SR^13^. As such, the observed delay of the nadir of the measured bulk SR Ca^2+^ depletion must be associated with the equilibration of these local Ca^2+^ gradients. Given the length of the delay between closure of the RyRs and the time to [Ca^2+^]_SR_ nadir, it is reasonable to suggest that there is a barrier to Ca^2+^ diffusion from the bulk SR to the near membrane as Ca^2+^ is released. This is likely to be calsequestrin (CSQ), given the positioning of CSQ at the SR terminal cisternae, immediately behind the RyRs and the unique Ca^2+^-adsorbing and high Ca^2+^-buffering capacity of CSQ^39–41^. Thus the spatiotemporal separation of Ca^2+^ sources inside the SR makes the near membrane a privileged signalling environment where Ca^2+^ can rapidly and locally deplete, with the opening and closing of RyRs. These transient depletions of Ca^2+^ near the membrane can happen without major depletions of the bulk [Ca^2+^]_SR_ be it during a spark, twitch or punctuated throughout a tetanus^13–15^. This system provides the environment for the rapid dissociation of Ca^2+^ from STIM1 in the near membrane^16^ for activation of SOCE following every action potential, as we have demonstrated (Figs. 1–4). Consistent with this, preliminary reports using Ca^2+^ indicators targeted close to the RyR channel within the SR terminal cisternae lumen have revealed that local Ca^2+^ release is greater and faster than that occurring in the bulk SR lumen during EC coupling^42^.

In respect thereof, the abundance and positioning of CSQ, RyRs and STIM1 in this environment are paramount to muscle Ca^2+^ homoeostasis and, ultimately, muscle physiology and health (e.g., refs. ^6,22,43–45^). While the normal positioning of CSQ and RyRs has been shown^39,46^, the exact positioning of STIM1 is unknown because of the lack of a specific STIM1 antibody to use for localization imaging^32^. Developing a picture of how STIM1 and RyRs position themselves relative to each other at the terminal SR and identifying how this arrangement changes under different conditions will be required to gain a further understanding of signalling at this nano-domain. However, our high-spatiotemporal imaging of Ca^2+^ transients (Figs. 1–4) during physiological Ca^2+^ release limits the possible location of the active proteins in the fibre to the junctional membranes, nearby the RyRs.

We propose that a primary physiological role of SOCE is an immediate, rapidly activated counter-flux to t-system Ca^2+^ extrusion in skeletal muscle fibres that is activated in response to the local depletion of SR terminal cisternae Ca^2+^ as evoked by action potentials. In the presence of a weakly Ca^2+^-buffered cytoplasmic environment, there was only a small net change in the [Ca^2+^]_t-sys_ during a train of action potentials (Fig. 6). This level of Ca^2+^-buffering is equivalent to the situation in the muscle, so allowing a build-up of Ca^2+^ at the cytoplasmic side of the t-system membrane during EC coupling to cause activation of Ca^2+^ transport from the junctional space to the t-system lumen. The simultaneous activation of SOCE with t-system Ca^2+^ extrusion represents an efficient mechanism to keep Ca^2+^ in the fibre during any period of stimulation. The advantage of the phasic Ca^2+^ influx being store-dependent is that the recovery of Ca^2+^ that enters the t-system during EC coupling is tightly regulated by the amount of Ca^2+^ exiting the SR, allowing balance of fluxes, or net Ca^2+^ influx that may assist muscle activity. Tracking the entry of radioactive Ca^2+^ into intact muscle during continuous, low-frequency stimulation has shown an accumulation of Ca^2+^ in the muscle^47^, suggesting that Ca^2+^ influx is greater than efflux in vivo^48^. The major component of this Ca^2+^ influx would be SOCE at the transverse tubules in conjunction with excitation (Figs. 1–4)^47^. The net movement of Ca^2+^ across the t-system is probably tuneable by regulation of the relative activity of the Na^+^–Ca^2+^ exchanger, plasma membrane CaATPase and SOCE. It remains possible that deviation from the Ca^2+^ homoeostasis we describe here may alter signalling cascades^10^.

Our demonstration that the normal activation of SOCE is immediate following every action potential-evoked release of Ca^2+^ in healthy muscle provides the framework in which to understand SOCE-related myopathies. e.g., ref. ^6^. It is not currently clear how quickly the absence of SOCE as a counter-flux will compromise fibre Ca^2+^ levels during bouts of EC coupling, as these fluxes are small (Fig. 4). Certainly, it is likely that changes in Ca^2+^ levels in the nano-domains of the junctional membranes during muscle activity will be sensitive to SOCE. The in-phase nature of the SOCE influx with the pattern of action potential stimulation (Figs. 1–4) also makes it possible that the SOCE transients entering the junctional space are an essential part of phasic Ca^2+^ signalling in response to muscle usage patterns^8–10,49,50^, as local Ca^2+^ signals activate diffusible messengers^51^.

Taken together, our results suggest the primary physiological role for SOCE in skeletal muscle to be a counter-flux to t-system Ca^2+^ extrusion, to maintain Ca^2+^ homoeostasis. Our observation of the control of t-system Ca^2+^-handling by action potential-evoked Ca^2+^ release can explain the classic observation of Armstrong et al.^52^, where skeletal muscle twitched for long periods in the presence of EGTA and virtual absence of external Ca^2+^. Under these conditions some of the Ca^2+^ released from SR is extruded across the t-system membrane to the t-system lumen. Ca^2+^ entering the t-system lumen requires seconds to diffuse from the fibre^53^. The simultaneous opening of the store-dependent channels with SR Ca^2+^ release (Figs. 1–4) turn the t-system into a sieve, causing the Ca^2+^ that entered the t-system lumen during Ca^2+^ release to re-enter the cytoplasm. That is, SOCE captures Ca^2+^ that is extruded to the t-system and returns it to the cytoplasm before it would otherwise be lost to the external environment beyond the t-system lumen.

## Materials and methods

### Preparation of skinned fibres and solutions for Ca^2+^ imaging

All experiments on rats were approved by the Animal Ethics Committee at The University of Queensland. Male Wistar and Sprague rats 4–8 months of age (University of Queensland Biological Resources, Brisbane) were killed by asphyxiation in CO_2_. The extensor digitorum longus (EDL) muscle was rapidly excised and placed in a Petri dish under paraffin oil above a layer of Sylgard. Small bundles of fibres were exposed to a Ringer solution containing 2.5 mM of the Ca^2+^-sensitive dye rhod-5N for at least 10 min. Thereafter, segments of individual fibres were isolated and the sarcolemma was mechanically removed with forceps (fibre skinning). The skinned fibres were mounted in a custom built chamber on top of a 1.5 coverslip and bathed in an internal solution (in mM): 90 HEPES, 10 EGTA, 40 HDTA (1,6-Diaminohexane-N,N,N′,N′-tetraacetic acid), 8.77 MgO, 1.94 CaCO_3_, 8 ATP*2Na, 10 creatine phosphate*2Na, with pH adjusted to 7.1 ± 0.1 with KOH and resulting free Ca^2+^ and Mg^2+^ concentration of 50 nM and 1 mM, respectively. Fluo-4 was added at a concentration of 10 µM. Electrical field stimulation was delivered by two platinum electrodes running in parallel to the fibre long axis. Individual pulses were applied at various frequencies (0.2–50 Hz) by rectangular voltage steps of up to 100 V in amplitude and 2–4 ms in duration.

The experimental chamber was mounted above a ×20 objective of a Leica TCS SP8 laser scanning confocal microscope system equipped with a 12 kHz resonant scanner. Rhod-5N and Fluo-4 were excited by 561 and 488 nm laser lines, respectively, and emitted light was collected with high-sensitivity GaAsP detectors in bands of 40 nm. xyt image series of the two colours were taken in sequential mode with an individual xy image size of 512 × 128 pixels and a time resolution of 18 ms (55 frames s^−1^). Pinhole was set to 1.5 AU. Fluorescence intensity across the imaged fibre area was averaged for both colours to derive traces of the mean fluorescence intensity over time that were then converted to [Ca^2+^]_cyto_ and [Ca^2+^]_t-sys_. Calcium concentrations ([Ca^2+^]), refer to free ion concentrations if not stated otherwise.

### Calibration of fluo-4 fluorescence and conversion to [Ca^2+^]_cyto_

The dissociation constant (*K*_*D*_) of fluo-4 was determined by measuring the dye fluorescence in the presence of free cytosolic [Ca^2+^] of 0.05, 0.8, 10 and 2000 µM in 4 fibres. Fluorescence values were normalized to the maximal values and fit with a Hill equation. The so obtained *K*_*D*_-value amounted to 1 µM, which is in good agreement with other reports^54^. Fluorescence recordings were then transformed to [Ca^2+^]_cyto_ according to the formula

1 $$\left[ {{\mathrm{Ca}}^{{\mathrm{2 + }}}} \right]_{{\mathrm{cyto}}}{ = K}_{D} \times \left( {{F - F}_{{\mathrm{min}}}} \right){\mathrm{/}}\left( {{F}_{{\mathrm{max}}}{ - F}} \right),$$

*F*_min_ was determined at the end of each experiment by exposing the fibre to a calcium free solution. *F*_max_ was calculated from the baseline fluorescence in the standard solution containing 50 nM of free [Ca^2+^] by rearranging equation (1):

2 $${F}_{{\mathrm{max}}} = {F} + \left( {{F} - {F}_{{\mathrm{min}}}} \right) \times {K}_{D}{\mathrm{/}}\left[ {{\mathrm{Ca}}^{2 + }} \right]_{{\mathrm{cyto}}}.$$

### Determination of the amount of total Ca^2+^ released from the SR during an electrically evoked action potential

This estimation is based on a method originally described by Melzer et al.^55^. Briefly, the cytosolic Ca^2+^ signals in 10 mM EGTA show a sharp transient followed by a slow decay upon each action potential (Fig. 1). The sharp transient is caused by the release of Ca^2+^ from the SR, which is incompletely buffered by EGTA while the slow decline reflects the diffusion of free Ca^2+^ out of the imaged region of interest, i.e., from within the fibre. Assuming the diffusive loss during the short time of an action potential to be negligible the change in cytosolic free Ca^2+^ levels before and right after an action potential directly reflects the additional amount of Ca^2+^ released from the SR. Considering a simple Ca^2+^-EGTA buffer system (Ca^2+^ +EGTA<–>Ca^2+^EGTA) and neglecting the much smaller endogenous buffering capacity^56^ the total amount of Ca^2+^ present can be calculated from the free [Ca^2+^]_cyto_ according to the following equation:

$$\left[ {{\mathrm{Ca}}^{{\mathrm{2 + }}}} \right]_{\mathrm{T}}{\mathrm{ = }}\left[ {{\mathrm{Ca}}^{{\mathrm{2 + }}}} \right] \times \left( {\left[ {{\mathrm{Ca}}^{{\mathrm{2 + }}}} \right]{ + K}_{D}{\mathrm{ + }}\left[ {{\mathrm{EGTA}}} \right]_{\mathrm{T}}} \right){\mathrm{/}}\left( {\left[ {{\mathrm{Ca}}^{{\mathrm{2 + }}}} \right]{ + K}_{D}} \right).$$

And the amount of total calcium released by the SR is obtained by:

3 $$\begin{array}{l}{\mathrm{\Delta }}\left[ {{\mathrm{Ca}}^{{\mathrm{2 + }}}} \right]_{\mathrm{T}}{\mathrm{ = }}\left[ {{\mathrm{Ca}}^{{\mathrm{2 + }}}} \right]_{{\mathrm{after}}} \times \left( {\left[ {{\mathrm{Ca}}^{{\mathrm{2 + }}}} \right]_{{\mathrm{after}}}{ + K}_{D}{\mathrm{ + }}\left[ {{\mathrm{EGTA}}} \right]_{\mathrm{T}}} \right){\mathrm{/}}\left( {\left[ {{\mathrm{Ca}}^{{\mathrm{2 + }}}} \right]_{{\mathrm{after}}}{ + K}_{D}} \right)\\ {\mathrm{- }}\left[ {{\mathrm{Ca}}^{{\mathrm{2 + }}}} \right]_{{\mathrm{before}}} \times \left( {{\mathrm{ }}\left[ {{\mathrm{Ca}}^{{\mathrm{2 + }}}} \right]_{{\mathrm{before}}}{ + K}_{D}{\mathrm{ + }}\left[ {{\mathrm{EGTA}}} \right]_{\mathrm{T}}} \right){\mathrm{/}}\left( {\left[ {{\mathrm{Ca}}^{{\mathrm{2 + }}}} \right]_{{\mathrm{before}}}{ + K}_{D}} \right),\end{array}$$

where [Ca^2+^]_before_ and [Ca^2+^]_after_ are the free calcium concentrations right before and right after each action potential, *K*_*D*_ is the dissociation constant of EGTA (200 nM)^56^, and [EGTA]_T_ is the total EGTA buffer concentration. The so converted values refer to the cytosolic volume of the fibre and were converted to values referring to the SR volume by assuming a cytosol to fibre volume ratio of 0.7 and a SR to fibre volume ratio of 0.093^57^.

4 $$\Delta \left[ {{\mathrm{Ca}}^{{\mathrm{2 + }}}} \right]_{{\mathrm{SR}}} = {\mathrm{\Delta }}\left[ {{\mathrm{Ca}}^{{\mathrm{2 + }}}} \right]_{\mathrm{T}} \times {\mathrm{7}}{\mathrm{.53}}.$$

### Calibration of t-system rhod-5N fluorescence

Calibration of rhod-5N fluorescence was performed as previously reported^19^. Fluorescence values were converted to [Ca^2+^]_t-sys_ using equation (1) with a *K*_*D*_ of 0.872 mM^19^.

### Determination of t-system depletion and recovery values

The change in [Ca^2+^]_t-sys_ (∆[Ca^2+^]_t-sys_ depletion) was calculated as the difference between the [Ca^2+^]_t-sys_ right before and immediately after each electrical stimulus. The recovery of [Ca^2+^]_t-sys_ (∆[Ca^2+^]_t-sys_ depletion) of the signal between consecutive stimuli was calculated as the difference between the [Ca^2+^]_t-sys_ right after and immediately before the following electrical stimulus.

### Determination of the relationship between ∆[Ca^2+^]_t-sys_ and ∆[Ca^2+^]_SR_

To derive the relationship between ∆[Ca^2+^]_t-sys_ and ∆[Ca^2+^]_SR_ (Fig. 4), [Ca^2+^]_t-sys_ depletion values and corresponding [Ca^2+^]_SR_ release values were determined under control conditions, in the presence of 10 µM of tetracaine, by back calculation in the presence of 30 µM of tetracaine, and for so called super events (see below). All of the so obtained values were then binned into the following intervals of ∆[Ca^2+^]_SR_: [0–0.5], [0.5–1], [1–2], [2–3], [3–4], [4–oo] mM. Data were fit with a nonlinear least square routine to a segmented equation consisting of an initial constant baseline followed by an exponential rise according to the formula:

5 $$\begin{array}{*{20}{l}} {{\mathrm{Y}} = {\mathrm{C}},} \hfill & {{\mathrm{for}}{\kern 1pt} 0 < {\mathrm{x}} < {\mathrm{x}}_0} \hfill \\ { = {\mathrm{C}} + {\mathrm{A}} \times \left( {1 - {\mathrm{e}}^{ - {\mathrm{k}} \times \left. {\left( {{x} - {x}0} \right)} \right)}} \right)} \hfill & {{\mathrm{for}}\,{x}> ={x}_0,} \hfill \end{array}$$

with *x*_0_ indicating the start of exponential rise.

### Back calculation of Ca^2+^ movements across the t-system membrane

Each action potential was accompanied by a depletion step, as well as a reuptake step. Upon repetitive stimulation [Ca^2+^]_t-sys_ eventually reached a new steady state, a condition in which the single depletion and reuptake steps must exactly balance each other (Supplementary Figure 2a). This relationship can be used to obtain the amplitude of the first depletion step under conditions in which the individual steps cannot be resolved anymore. The change in t-system steady-state Ca^2+^ level (∆[Ca^2+^]^SS^_t-sys_) during repetitive stimulation is the sum of all individual depletion and re-uptake steps, i.e.

6 $${\mathrm{\Delta }}\left[ {{\mathrm{Ca}}^{{\mathrm{2 + }}}} \right]_{{\mathrm{t - sys}}}^{{\mathrm{SS}}} = {{\sum_{\mathrm i}^\infty}}\left( {{\mathrm{Yd}}_{i}-{\mathrm{ Yr}}_{i}} \right),$$

with Yd_*i*_ and Yr_*i*_ being the *i*^th^ amplitude of depletion and re-uptake, respectively. The amplitudes of the individual depletions steps followed an exponential, while the amplitudes of the reuptake remained constant (Supplementary Figure 2a), thus

7 $$\begin{array}{l}{\mathrm{Yd}}_{i}{\mathrm{ = }}\left( {{\mathrm{Y}} - {\mathrm{C}}} \right) \times {\mathrm{e}}^{ - {\mathrm{k}} \times {i}}\\ {\mathrm{Yr}}_{i} = {\mathrm{C}}\end{array}.$$

Summing of the geometric series as obtained by inserting expression (7) into (6) yields an expression for the first depletion step in a train of stimulation as a function of ∆[Ca^2+^]^SS^_t-sys_:

8 $${\mathrm{Y = \Delta }}\left[ {{\mathrm{Ca}}^{{\mathrm{2 + }}}} \right]^{{\mathrm{SS}}}_{{\mathrm{t - sys}}} \times \left( {{\mathrm{1 - e}}^{{\mathrm{ - k}}}} \right){\mathrm{ + C}}.$$

### Super events

On a few occasions electrical stimulation did not result in the generation of only a single action potential but was accompanied by a second action potential or even a small burst like discharge. Also, spontaneous action potentials occasionally seen during experiments happened to sometimes coincide with the electrical stimulation. Although these super events released considerably larger amounts of Ca^2+^ into the cytosol as determined by the fluo-4 signal the respective depletion of the t-system was only moderately increased reaching a limiting level of about 0.3 mM.

### Statistical analysis

For comparing two groups of data a two-tailed Student’s *t*-test was used. For comparing more than two groups ANOVA with Tukey’s post hoc test was used. Paired testing was applied when the data points to compare were sampled from the same fibres. All data are given as mean ± SEM.

### Data availability

The authors declare that all data supporting the findings of this study are available within the article and its supplementary information files.

## Electronic supplementary material

Supplementary Information(PDF 1903 kb)
