# Supplementary material for: Store-operated Ca2+ entry is activated by every action potential in skeletal muscle
Source: Commun Biol. 2018 Apr 19;1:31. doi: 10.1038/s42003-018-0033-7 (PMC6123720; doi:10.1038/s42003-018-0033-7)
Supplement: Supplementary file 1 — Supplementary Information(PDF 1903 kb) [file 42003_2018_33_MOESM1_ESM.pdf]

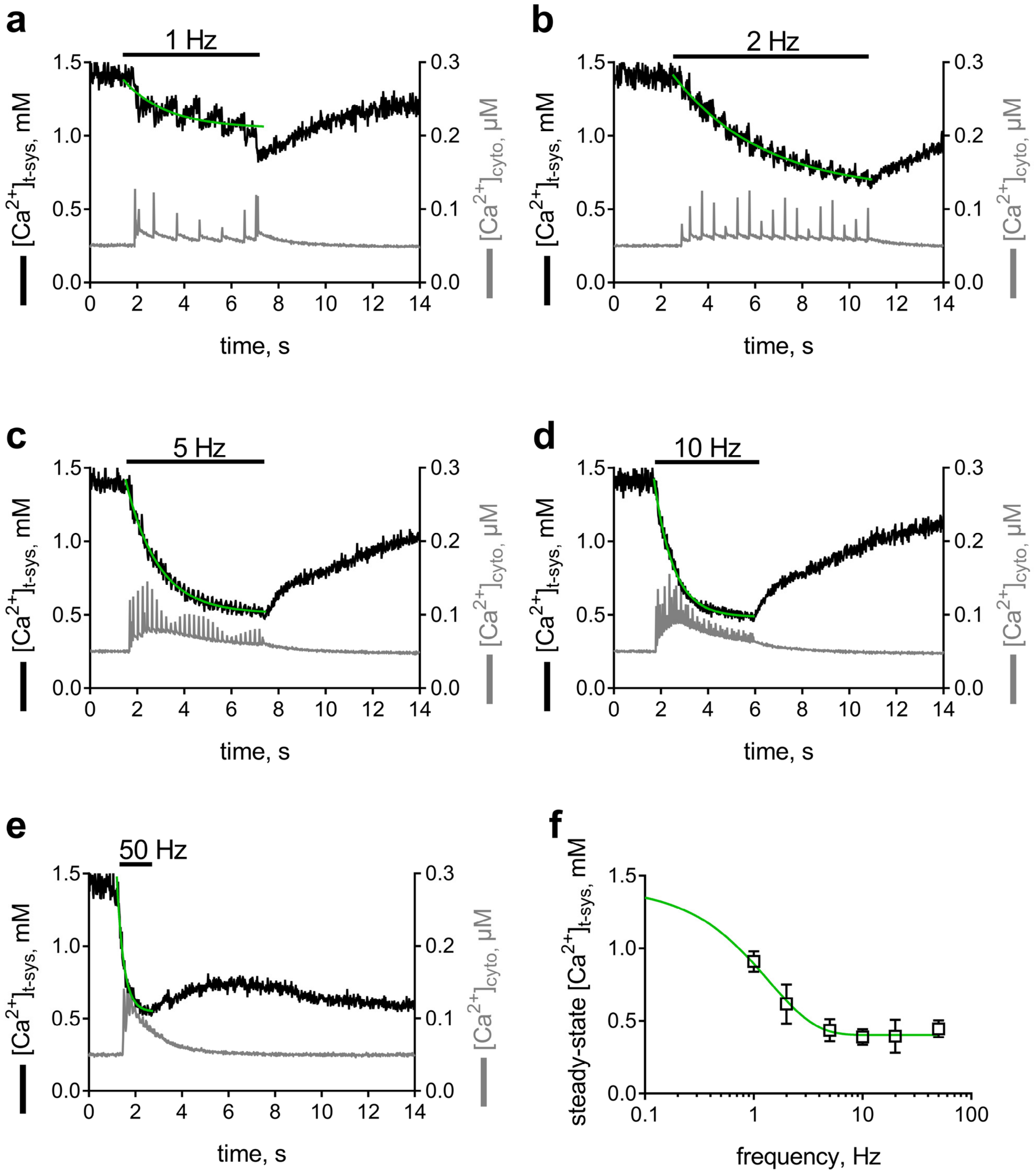

Supplementary Figure 1. Depletion of the t-system is associated with action potential frequency. (a) Typical recordings of $[Ca^{2+}]_{t\text{-}sys}$ (left axes) and $[Ca^{2+}]_{cyto}$ (right axes) over time as derived from skinned rat EDL fibres during electrical stimulation. xyt image series of the fluorescent signals of rhod-5N and fluo-4 trapped in the t-system and loaded into the cytosol, respectively, were spatially averaged and calibrated (Methods). Electrical stimulation at 1, 2, 5, 10 and 50 Hz as indicated was started shortly after beginning of the recording and was continued until a new steady state in the t-system was reached. (b) Summary of the nadir steady state level $[Ca^{2+}]_{t\text{-}sys}$ reached for different stimulation frequencies. Data are derived from 10 fibres and given as mean ± SEM.

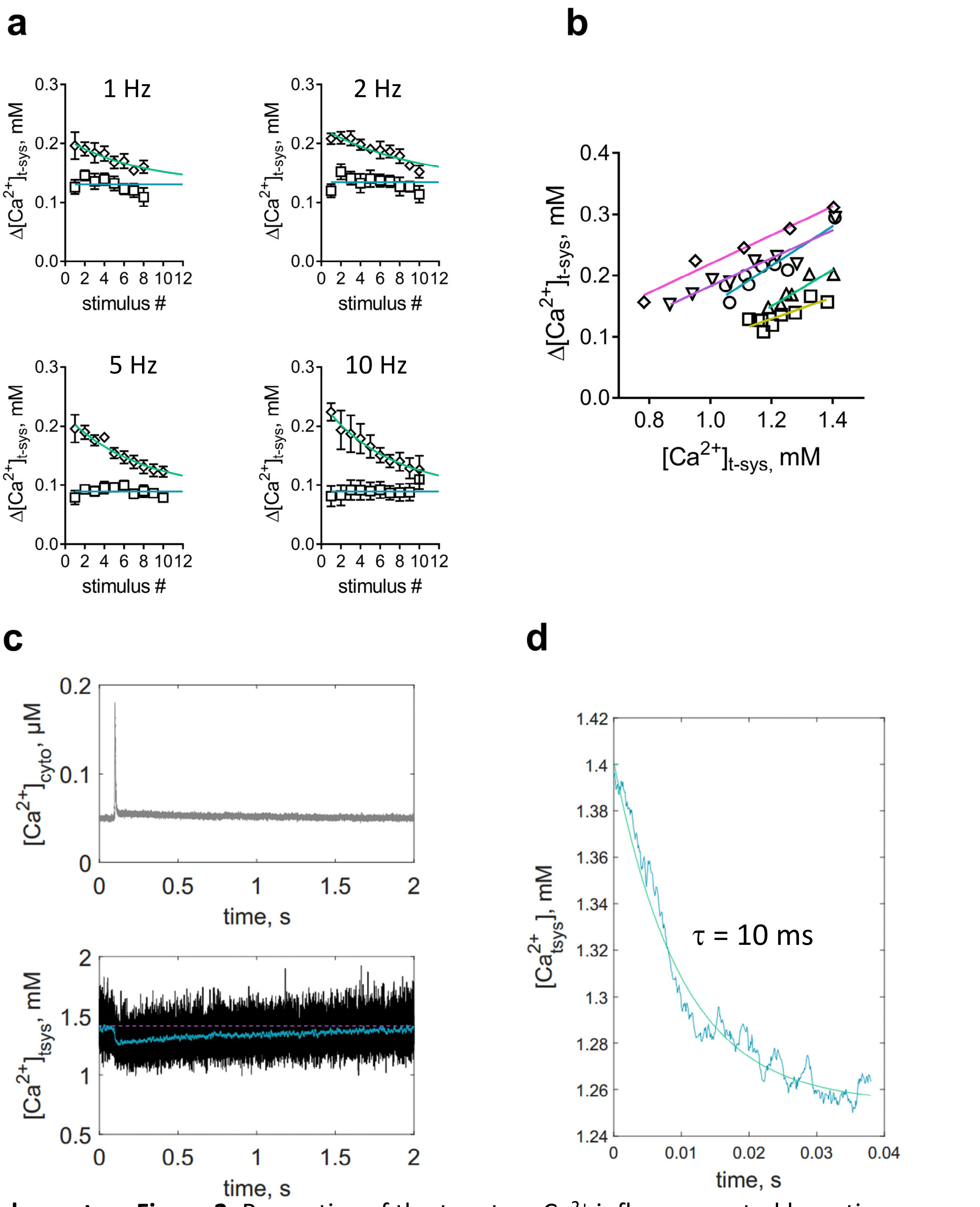

Supplementary Figure 2. Properties of the t-system Ca^{2+} influx generated by action potentials. (a) Depletion (diamonds) and recovery of (squares) $[Ca^{2+}]_{t\text{-}sys}$ for each stimuli during trains of APs at the indicated frequency. (b) $\Delta[Ca^{2+}]_{t\text{-}sys}$ depletion from an initial $[Ca^{2+}]_{t\text{-}sys}$ following AP-evoked Ca^{2+} release derived from 5 independent experiments, indicated by the differently coloured lines. (c) The $[Ca^{2+}]_{cyto}$ (top graph) and $[Ca^{2+}]_{t\text{-}sys}$ (bottom graph) during AP stimulation recorded at 80 µs $line^{-1}$. The original $[Ca^{2+}]_{t\text{-}sys}$ trace is in black and the filtered signal is in turcoise (bottom graph). The pink dotted line indicates the original level of $[Ca^{2+}]_{t\text{-}sys}$. (d) The filtered $[Ca^{2+}]_{t\text{-}sys}$ signal during the decline due to SOCE activation could be fitted by an exponential function with a time constant of 10 ± 1 ms (mean ± SEM, n=4).

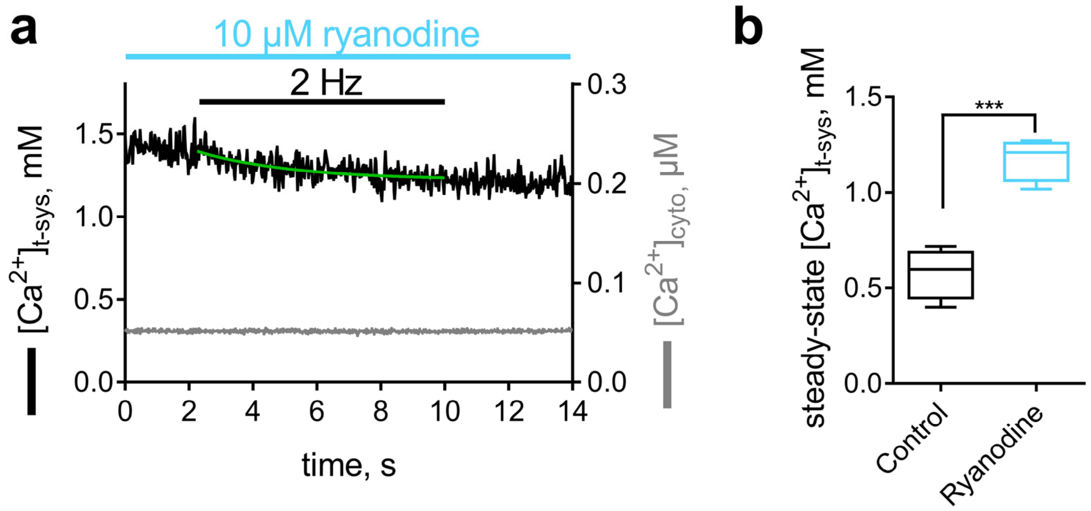

Supplementary Figure 3 The RyR antagonist ryanodine inhibits AP-evoked Ca^{2+} release and SOCE. (a) Trace of $[Ca^{2+}]_{t\text{-}sys}$ and $[Ca^{2+}]_{cyto}$ during 2 Hz electrical stimulation of a skinned fibre in the presence of 10 µM ryanodine. Note that Ca^{2+} release from the SR was blocked and no phasic depletions of $[Ca^{2+}]_{t\text{-}sys}$ were observed. A tonic depletion of $[Ca^{2+}]_{t\text{-}sys}$ was still observed during electrical stimulation. (b) Summary of steady-state $[Ca^{2+}]_{t\text{-}sys}$ at the end of the 2 Hz stimulation in the absence and presence of 10 µM ryanodine from a total of n = 3 fibres, presented as a box and whisker plot**.** Paired two-tailed Student's t-test . *** indicates statistical significance with p=0.0002.

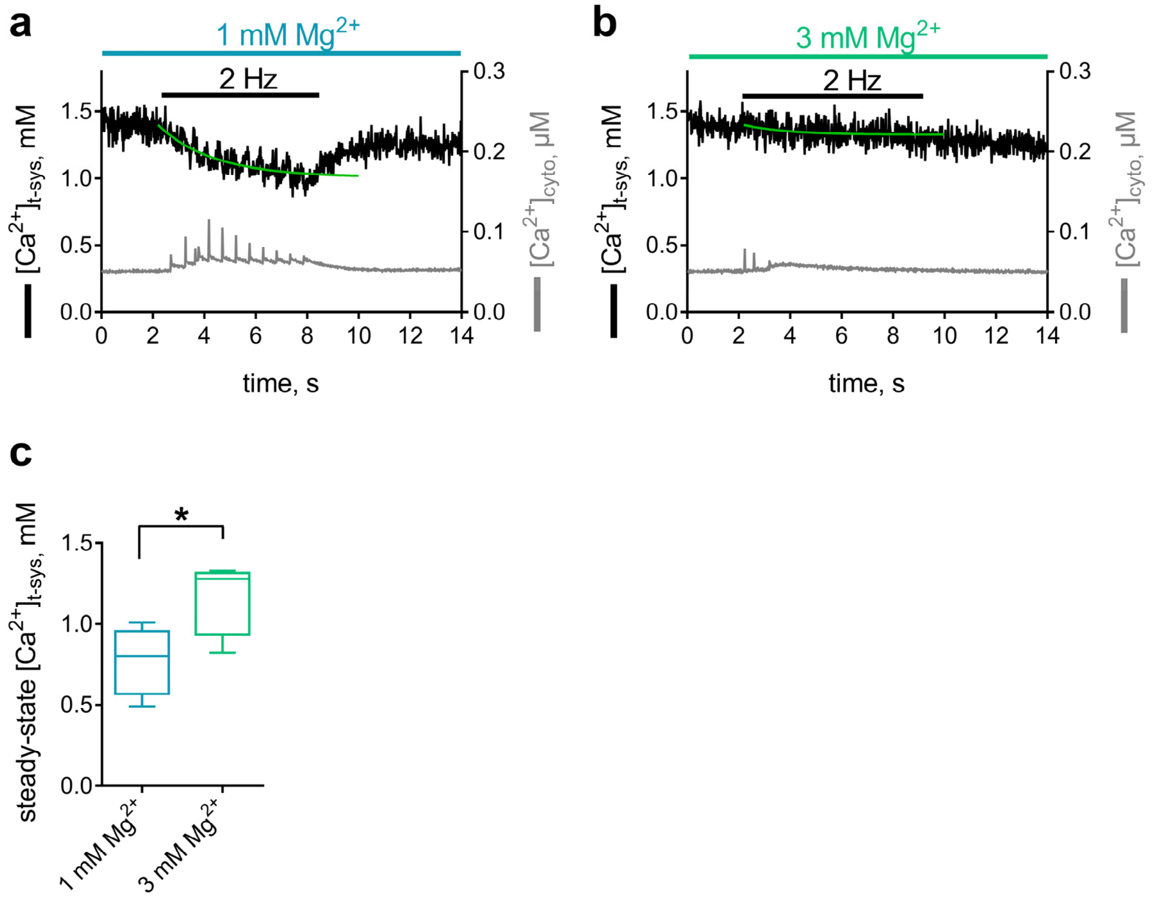

Supplemental Figure 4 Raised $[Mg^{2+}]_{cyto}$ inhibits AP-evoked Ca^{2+} release and SOCE. (a) Trace of $[Ca^{2+}]_{t\text{-}sys}$ and $[Ca^{2+}]_{cyto}$ during 2 Hz electrical stimulation of a skinned fibre in the presence of 1 (a) and 3 (b) mM $[Mg^{2+}]_{cyto}$. Note that Ca^{2+} release from the SR was almost abolished and no phasic depletions of $[Ca^{2+}]_{t\text{-}sys}$ were observed in the presence of 3 mM Mg^{2+}. A tonic depletion of $[Ca^{2+}]_{t\text{-}sys}$ was still observed during electrical stimulation. (c) Summary of steady-state $[Ca^{2+}]_{t\text{-}sys}$ at the end of the 2 Hz stimulation in the presence of 1 and 3 mM Mg^{2+} from a total of n = 4 fibres presented as a box and whisker plot**.** Paired two-tailed Student's t-test . * indicates statistical significance with p=0.0183.

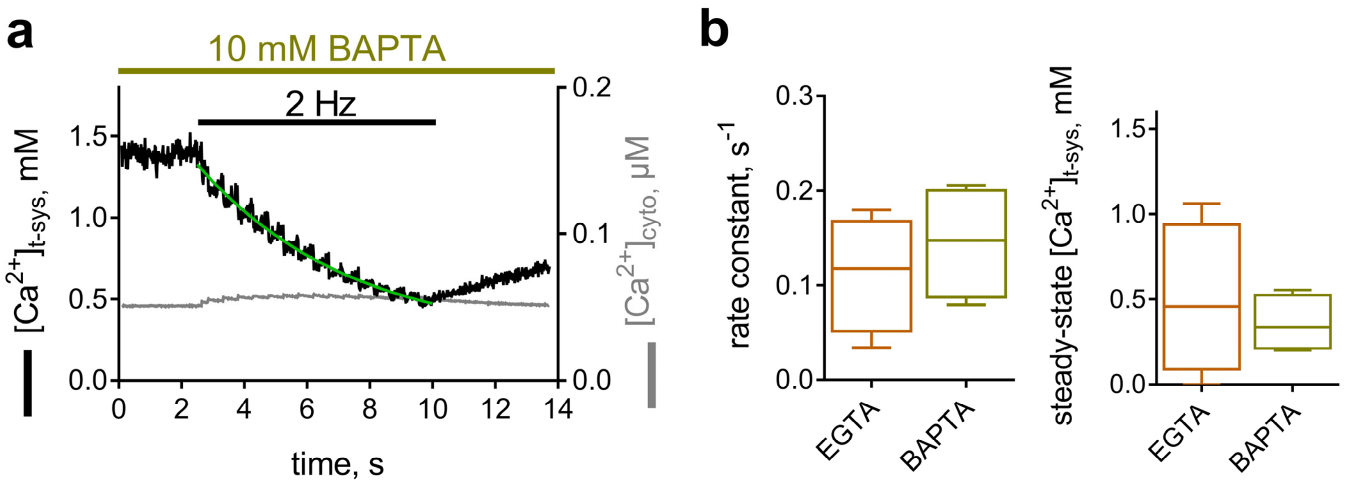

Supplemental Figure 5 The effect of EGTA and BAPTA on the $[Ca^{2+}]_{t\text{-}sys}$ transient during electrical stimulation. (a): In the presence of 10 mM Bapta during 2 Hz electrical stimulation the spikes in the cytoplasmic Ca^{2+} transient are not observed because of Bapta is rapid enough to buffer this rapid Ca^{2+} transient that is seen in EGTA. This confirms that the $[Ca^{2+}]$ in the junctional space during EC coupling will be different in the presence of EGTA and BAPTA. Note that the 2 Hz stimulation still causes phasic t-system Ca^{2+} influx to deplete $[Ca^{2+}]_{t\text{-}sys}$. (b): the rate constant and lower plateau of $[Ca^{2+}]_{t\text{-}sys}$ depletion. The result is expressed as a box and whisker plot from 4 fibres. Two-tailed Student's *t*-tests revealed no significant differences for rate constants and steady-state $[Ca^{2+}]_{t\text{-}sys}$ (p = 0.4728 & 0.5833, respectively).

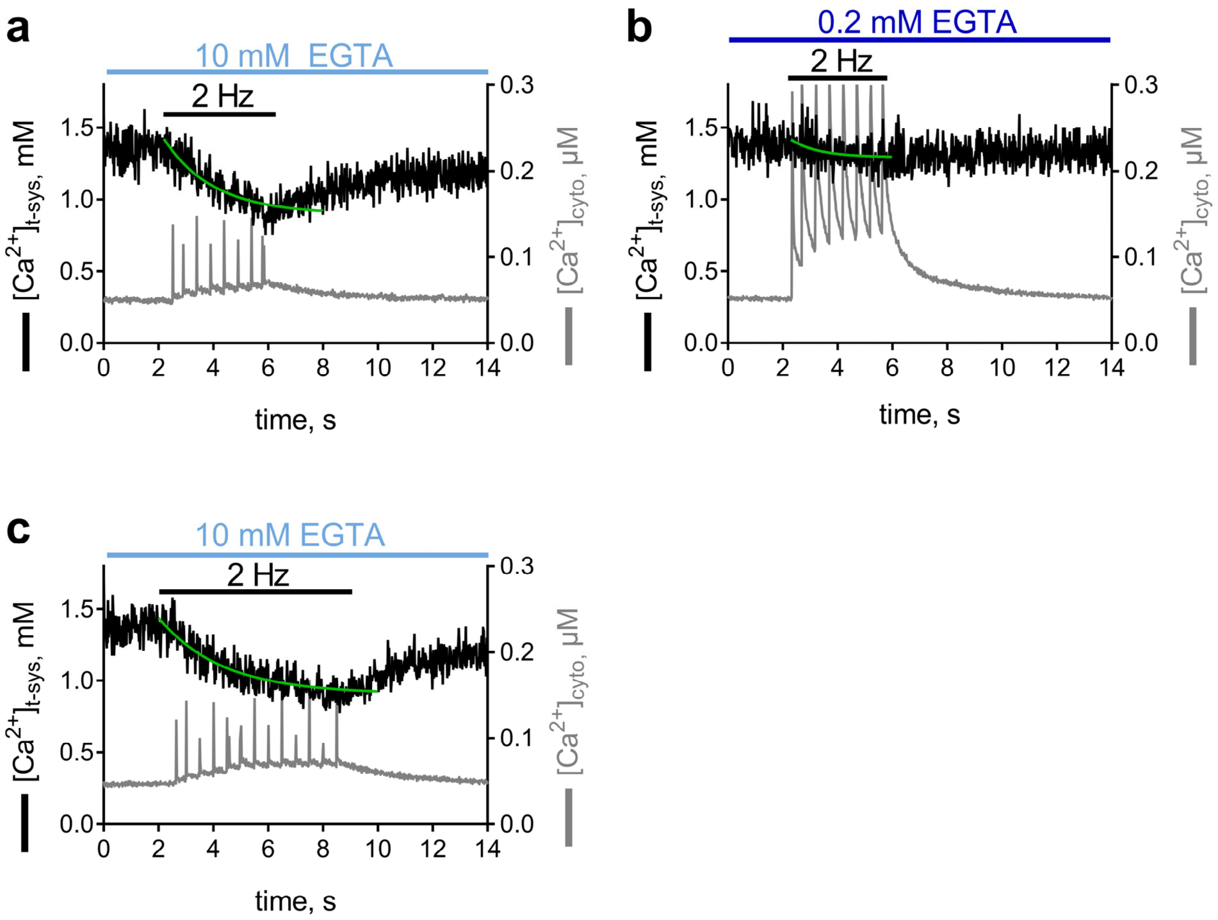

Supplemental Figure 6 The effect of changing cytoplasmic Ca^{2+}-buffering on the $[Ca^{2+}]_{t\text{-}sys}$ transient during electrical stimulation is reversible. In the same fibre, the presence of 10 mM EGTA (a) allowed 2 Hz stimulation to deplete $[Ca^{2+}]_{t\text{-}sys}$. Replacing the cytoplasmic solution with 0.2 mM EGTA and applying the same stimulation protocol (b) did not result in a resolvable depletion in $[Ca^{2+}]_{t\text{-}sys}$. Returning the fibre to 10 mM EGTA (c) again allowed a resolvable depletion in $[Ca^{2+}]_{t\text{-}sys}$ during stimulation.
